# Pre-clinical models to define correlates of protection for SARS-CoV-2

**DOI:** 10.3389/fimmu.2023.1166664

**Published:** 2023-03-30

**Authors:** Caolann Brady, Tom Tipton, Stephanie Longet, Miles W. Carroll

**Affiliations:** ^1^ Nuffield Department of Medicine, Wellcome Centre for Human Genetics and Pandemic Sciences Institute, University of Oxford, Oxford, United Kingdom; ^2^ International Center for Infectiology Research (CIRI), Team GIMAP, Claude Bernard Lyon 1 University, Inserm, U1111, CNRS, UMR530, Saint-Etienne, France

**Keywords:** animal models, SARS-CoV-2, vaccines, immunity, correlates of protection (CoP)

## Abstract

A defined immune profile that predicts protection against a pathogen-of-interest, is referred to as a correlate of protection (CoP). A validated SARS-CoV-2 CoP has yet to be defined, however considerable insights have been provided by pre-clinical vaccine and animal rechallenge studies which have fewer associated limitations than equivalent studies in human vaccinees or convalescents, respectively. This literature review focuses on the advantages of the use of animal models for the definition of CoPs, with particular attention on their application in the search for SARS-CoV-2 CoPs. We address the conditions and interventions required for the identification and validation of a CoP, which are often only made possible with the use of appropriate *in vivo* models.

## 1 Introduction

The outbreak of a novel coronavirus, subsequently named severe acute respiratory coronavirus 2 (SARS-CoV-2), in Wuhan, China, in early December 2019, rapidly escalated to pandemic status on March 11^th^ 2020 (1). SARS-CoV-2, the causative agent of coronavirus disease, COVID-19, has since claimed over 6 million lives (2). Fortunately, the development, approval and deployment of effective SARS-CoV-2 vaccines has since significantly reduced fatality rates and ameliorated the impact of SARS-CoV-2 infections on billions of lives.

Since the beginning of the pandemic, international efforts have been made to understand SARS-CoV- 2 pathogenicity and host immune responses. However, the immune profile/s associated with protection is/are still unclear. A person’s immunity to a pathogen can be inferred by measurement of the component of the “immune response that is responsible for and statistically interrelated with protection,” also known as a ‘correlate of protection (CoP)’, a term coined by Stanley Plotkin (3). Profiling the prevalence of a CoP in community samples of vaccinees and convalescents would efficiently capture rates of immunity against the disease of interest. Hence, a CoP is a parameter that is invaluable for the fields of vaccinology, infectious disease immunology, epidemiology and health policy.

To define a CoP unequivocally, animal research remains critical - from the early stages of understanding the immunology of a disease, to providing proof-of-concept evidence in support of candidate CoPs. SARS-CoV-2 is no different, with COVID-19 animal models being central to the extraordinary immunology research and vaccine development efforts over the last three years. This review focuses on the mechanisms by which SARS-CoV-2 animal research assists, and in some cases supersedes, human research in the search for SARS-CoV-2 CoPs, with reference to the immunological insights provided by COVID-19 animal models used in challenge and vaccine pre-clinical studies.

## 2 Correlates of protection

Identifying a CoP that is universally observed and easily measured in an immunised population is of significant value for; a) vaccine development, such that vaccine candidates aim to drive the protective response, b) vaccine licensure, whereby protection likelihood can be inferred upon measurement of a CoP in vaccinees (also known as immunobridging), and c) public health policy-making in the midst of a pandemic, where accurate rates of immunity can be measured to appropriately inform governmental decisions on the application of non-pharmaceutical interventions (NPIs). A prime example of a validated CoP that supports vaccine development is haemagglutinin inhibition (HAI) titres (of at least 1:40) for the approval of seasonal influenza vaccines (4).

For the purpose of this review, it is important to consider precisely what ‘protection’ means in the context of SARS-CoV-2 infection, particularly as COVID-19 has many variable manifestations in humans, including asymptomatic, mild, moderate, severe or fatal disease, and chronic disease. Successful management of a pandemic relies on reduced transmission rates and/or lower rates of hospitalisation, therefore either low virology scores or minimal pathology could be considered as a ‘protected’ outcome. This review focuses on animal models where veterinary pathologist assessments, post-cull, engender the scope for precise characterisation of clinical as well as virology outcomes post-challenge. This is particularly valuable as the mechanism of limiting viral load may differ from the mechanism of infection control and resolution.

The terms ‘co-correlate’ and ‘surrogate’ describe a collaborative and redundant network of protective immune mechanisms, respectively (3). ‘Absolute’ and ‘relative’ correlates capture the weight of involvement of the immune parameter in mediating protection, either by dominating the protective response (the former) or by variably contributing to protection (the latter) (3). In the search for a CoP, knowledge gaps must also be accounted for; a statistically significant CoP may, in actuality, be a by-product of an unknown protective mechanism. We need to recognise the limitations and shortcomings of statistical correlations and that even with supporting ‘proof-of-concept’ investigations, such as adoptive transfer or depletion experiments, unknown knock-on effects of such immune manipulations may contribute to the observed exacerbation or elimination of infection.

Additionally, given immune system variability with age, sex, genetics, epigenetics and microbiome composition, a CoP in one population may differ to that in another population, particularly with respect to the magnitude of responses and epitope-specific responses. In which case, a universal CoP for SARS-CoV-2 may not exist, with different parameters defining protection in different groups. Hence, it is important to bear in mind which populations are involved in the study and whether data from different cohorts is pooled for analysis in an attempt to extract a universal CoP, or alternatively the data is separated by age, sex or ethnicity to define cohort-specific CoPs. Furthermore, each vaccine platform or formulation may exploit different protective immune mechanisms, thereby pointing to the existence of vaccine-specific CoPs. Such factors convolute the definition of a CoP.

## 3 COVID-19 animal models

**Figure 1:**
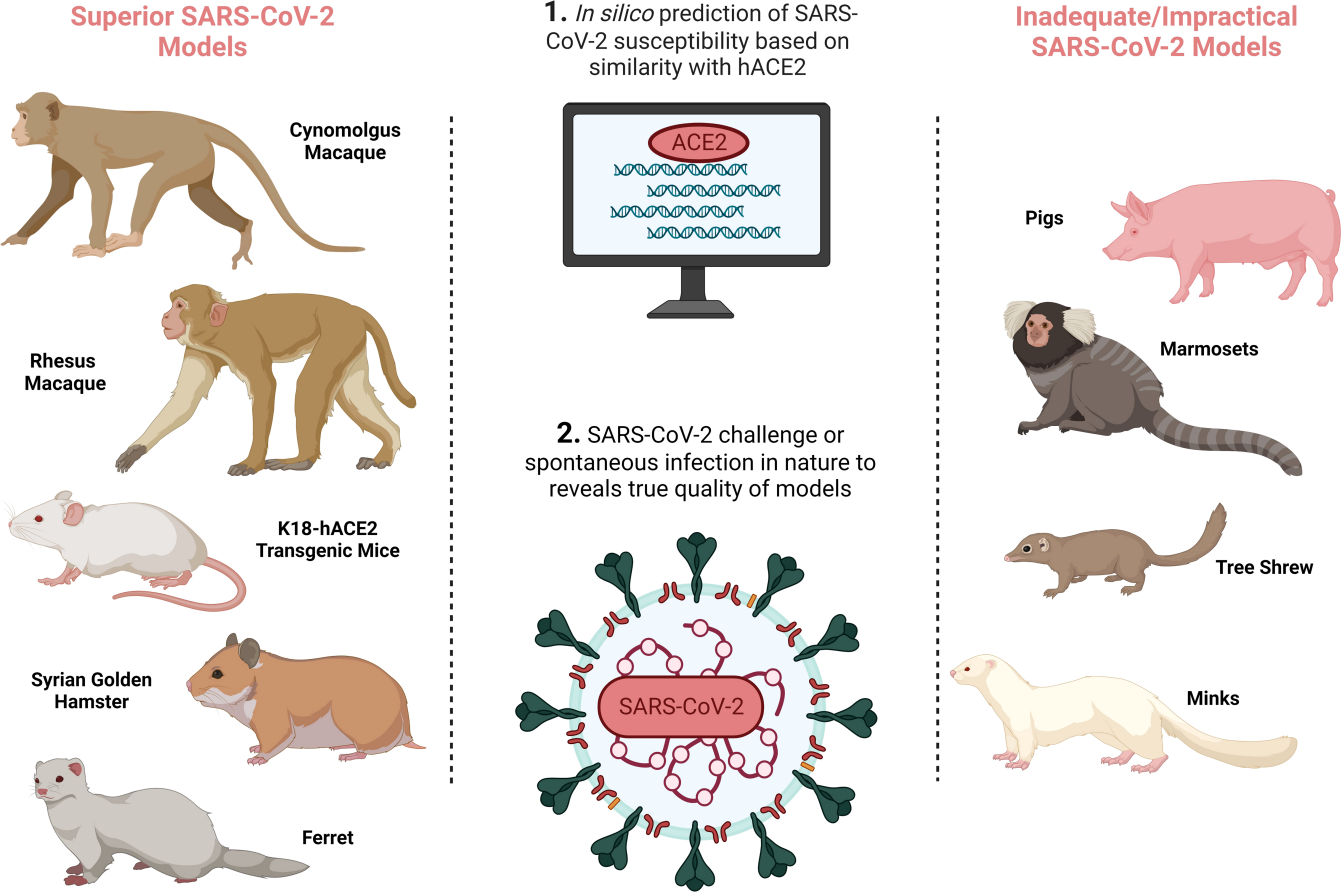
SARS-CoV-2 animal model development overview demonstrating the preferred SARS-CoV-2 animal models on the left, and inadequate/impractical COVID-19 models with defined limitations on the right, and the processes by which animal models are identified in the central column. Created with BioRender.com.

It is essential that CoP research is based on authentic, relevant and reproducible *in vivo* models. Since the emergence of SARS-CoV-2 in December 2019, significant efforts have been made to develop accurate COVID-19 animal models (as extensively reviewed in (5–7) and summarised in **Figure 1** and **Table 1**) to probe and understand COVID-19 pathology and immunology in order to develop effective therapeutics and vaccines. Given how critical an animal model is for research into a novel, pandemic-causing virus, the World Health Organisation (WHO) Research and Development Blueprint Team launched WHO-COM, a group focused on ‘COVID-19 *in vivo* modelling’ (55), as has the National Institute of Health (NIH) through the ‘Accelerating COVID-19 Therapeutic Interventions and Vaccines’ (ACTIV) preclinical working group.’ The WHO recognises non-human primates (NHPs), ferrets, mice and hamsters, to differing degrees, as models that capture the major physical manifestations of COVID-19, including pathology and/or immune signatures.

**Table 1:** Summary of the pros and cons associated with each model and the research papers discussed in this review that applied these models for the definition of CoPs.

Animal	Pros	Cons	Is the model in use?	COVID-19 Research References
**Rhesus Macaque**	* Captures Mild-Moderate COVID-19 lung pathology (8) * Old RhMs Model Severe COVID-19 (8) * 94% Genetic Similarity to Humans	* Shortages (8) * Ethical Issues * Housing requirements are costly	Yes	(8–28)
**Cynomolgus Macaque**	* Captures Mild-Moderate COVID-19 (8) * Restricted HLA (8)	* Lung Cell ACE2 Expression Patterns Differ to Humans (29) * Ethical Issues * Housing requirements are costly	Yes	(8, 30, 31)
**Syrian Golden Hamster**	* Small * Models Severe COVID-19 when Challenged with High Doses (32) * Captures age-bias of COVID-19 (33)	* Lack of Hamster Reagents * Lung Cell ACE2 Expression Patterns Differ to Humans (34)	Yes	(12, 13, 32, 33, 35–40)
**K18-hACE2 C57BL/6 and hACE2 BALB/c Mice**	* Small * Manipulate Genetics * Captures Clinical COVID-19 Symptoms (41)	* Inter-Mouse Variability of hACE2 Expression * High Viral Load in the Brain (42)	Yes	(40, 41, 43–47)
**Ferret**	* Susceptible to SARS-CoV-2 Infection * Captures Mild Clinical Symptoms (41, 48)	* Predominantly Models URT Infection (41)	Yes	(21, 23, 49)
**Mink**	* Susceptible to SARS-CoV-2 Infection (41)	* Lab Handling Difficulties	TBD	
**Marmoset**	* Susceptible to SARS-CoV-2 Infection	* Fail to Mount IgG Response (50)	No	
**Tree Shrew**	* Susceptible to SARS-CoV-2 Infection	* No SARS-CoV-2 Shedding (51) * Only Clinical Symptom is Body Temperature Changes (51) * Inverse age bias (52)	No	
**Pig**	* Strong similarities with human anatomy, physiology and immunology	* No Affinity between Swine ACE2 and Spike Protein * Not Susceptible to SARS-CoV-2 Infection (53) (54)	No	

The genus of Old World Monkeys (OWMs) (taxonomically known as *Cercopithecidae*), are the gold standard for modelling human disease, given the extensive homology between the closely-related species that diverged twenty-three to twenty-five million years ago. The Rhesus Macaque Genome Sequencing and Analysis Consortium reported that macaques and humans share 93.54% sequence identity, with 97.5% sequence identity in high confidence orthologues (56). Salguero et al. has provided a direct comparison of the key macaque species used in COVID-19 research, rhesus macaques (*Macaca mulatta*) (RhMs) and cynomolgus macaques (*Macaca fascicularis*) (CyMs). Matched viral strains, viral loads and routes of challenge were used to conclude that RhMs and CyMs have a similar capacity to recapitulate a mild-to-moderate human SARS-CoV-2 infection, both pathologically, virologically and immunologically (8).

BALB/c and C57BL6 mice also model COVID-19, with the added advantages of housing- and manipulation-ease. They share 69.1% sequence identity with humans. Murine angiotensin-converting enzyme 2 (ACE2), which shares 82.11% of its gene sequence with humans, does however have negligible affinity to SARS-CoV-2 Spike (57). Thus, the murine SARS-CoV-2 infection model requires either genetic manipulation of the SARS-CoV-2 virus so as to become mouse-adapted, or the breeding of transgenic mice that express human ACE2 (hACE2) e.g. K18-hACE2 C57BL/6J mice (a flaw of which is the variability of hACE2 expression levels) (7). It is important to note that cause of death in mice following SARS-CoV-2 infection is high viral burden in the brain rather than in the lung, implying COVID-19 pathology differs between the species (42). However, these models do capture human COVID-19 clinical symptoms of anosmia, thrombosis, and weight loss (41).

Syrian Hamster ACE2 has affinity to SARS-CoV-2 Spike and hence the species are naturally susceptible to SARS-CoV-2 infection (41). Clinical symptoms consistent with COVID-19 are seen, including weight loss, respiratory distress and inflammation-driven lung pathology (41). For example, a 5% increase in percentage weight loss as a consequence of high dose challenge was reported in Syrian hamsters, with 75% of these animals meeting the humane euthanasia criteria by day seven post-challenge, thus capturing severe COVID-19 (32). Furthermore, this small animal model mirrors the age-bias of COVID-19, with thirty-two to thirty-four-week-old hamsters exhibiting more substantial weight loss during acute infection, as well as persistent inflammation and unresolved lung tissue damage fourteen days post-challenge with SARS-CoV-2, in comparison to the six-week-old hamsters that had recovered by this timepoint (33).

Ferrets are susceptible to SARS-CoV-2 infection as evident from the mild lung pathology and detection of viral shedding in the nose and throat (41, 48). SARS-CoV-2 is detectable in the upper respiratory tract (URT) of ferrets by day two post-challenge and they present with similar URT symptoms to humans, including nasal discharge and sneezing (41).

Inadequate/impractical SARS-CoV-2 infection models include pigs, where despite *in silico* prediction of swine ACE2 and SARS-CoV-2 Spike affinity, supported by their natural susceptibility to SARS-CoV-1, they are poor SARS-CoV-2 hosts. Minks on the other hand are susceptible to SARS-CoV-1 and SARS-CoV-2 with outbreaks of the latter occurring in mink farms (58), however, their aggressive nature restricts their use as a common laboratory model (41).

## 4 Limitations of human studies for the definition of SARS-CoV-2 CoPs that are met by animal models

Prior to the emergence of effective vaccines, human largescale serology studies reported that SARS-CoV-2-specific immune responses were detectable following primary exposure, which conceptually could provide protection upon re-exposure (59–64). However, the degree of protection in the studied populations was heterogenous [further complicated by variants of concern (VOCs)]. Identifying the role of the humoral and/or the cellular response in mediating protection, as well as the precise characteristics of the humoral and/or cellular response that engender this protection, remains a challenge. *In vivo* models have played a key role in pin-pointing which immune parameters are mediating this protection in humans.

A major limitation of human studies for the identification of CoPs, is the failure to conclude, with confidence, whether the anamnestic response following natural primary infection or vaccination is protective upon re-exposure. Firstly, researchers cannot say with certainty whether or not a convalescent or vaccinated participant has been exposed to the pathogen during the follow-up period, particularly if an individual were to have sterilising immunity against the pathogen and would therefore lack any serological evidence of breakthrough/reinfection (an anti-SARS-CoV-2 Nucleocapsid response would only emerge post-infection). Vaccine efficacy is deduced from the reduced rates of infection seen in vaccinees versus placebo controls in an area where the virus is endemic and in active circulation during the clinical trials. However, vaccinees are not necessarily ever exposed to the aetiological agent during the course of the trial. Government-enforced lockdowns and other NPIs in place during the phase II and III clinical trials would no doubt impact the rate of transmission, which firstly, may disguise the true weight of impact of vaccination while also limiting the number of infections, thus restricting the statistical significance of outcomes. Swadling et al. did attempt to ascertain exposures to SARS-CoV-2, using IFI27 as a blood biomarker of viral exposure at subclinical levels, such that PCR negativity and IFI27 positivity would suggest protection from reinfection (59, 65, 66). However, it is important to note that IFI27 is non-specific for SARS-CoV-2, and is upregulated in response to other respiratory infections including H1N1/09 influenza and respiratory syncytial virus (66). In which case, it must be recognised that only the endpoint of failed protection can be confidently defined in humans, with the eventuality of a positive PCR result post-vaccination or primary infection, while the endpoint of successful protection is largely ambiguous.

The incidence of asymptomatic COVID-19 further limits our ability to characterise successful protection. Rates of asymptomatic SARS-CoV-2 infections are high, with 47% of 165 SARS-CoV-2 positive cases in a ~12,000 frontline worker cohort reported as asymptomatic (64). PCR testing was predominantly encouraged for those presenting with COVID-19 symptoms in large-scale human studies, therefore endpoints of failed protection in the form of asymptomatic COVID-19 often fail to be recorded. The consequence of failing to capture asymptomatic cases is seen in the phase III trial of mRNA-1273. mRNA-1273 was reported to be 94.1% effective in preventing COVID-19 from fourteen days post-boost, however, this trial only identified symptomatic COVID-19 cases, given the limited capacity for routine screening of the large population size (n = ~30,000) (67). The revised efficacy of mRNA-1273 was subsequently found to be 82% when asymptomatic cases were accounted for (68). Failing to accurately capture asymptomatic cases also limits the drawing of associations between vaccine-induced immune responses and protection against asymptomatic infection, as was unsuccessful in the study by Feng et al. (69).

Inherent human biases have also been shown to skew PCR testing frequency. In a study by Lumley et al., baseline seropositive and seronegative healthcare workers (HCWs) were screened biweekly in an attempt to define an association between the rates of PCR-positivity and anti-Spike IgG titres. The group observed that seronegative HCWs had a much greater attendance to the non-obligatory asymptomatic screenings than seropositive individuals, epitomising a phenomenon known as ‘outcome ascertainment bias,’ which manifests in convalescent participants with an inherent assumption that reinfection is unlikely. The problem with bias in this particular setting, is that over the course of this investigation, seropositive HCWs that did return PCR-positive results during mandatory testing presented with asymptomatic infections, further increasing the likelihood that there were undetected PCR-positive results amongst the seropositive HCWs (64). Testing of human participants must therefore be frequent and mandatory, irrespective of symptom presentation, if researchers carrying out vaccine efficacy studies wish to detect near 100% of infections.

High variability between human re-exposure events is also inevitable in a real-world setting. This includes variable viral inocula which will have an impact on whether or not COVID-19 disease manifests, and cannot be accounted for when comparing human vaccinees and control groups. Furthermore, given the rapid evolutionary trajectory of a virus upon zoonosis to a new host species, it is possible that human participants are exposed to different viral strains. In fact, reinfection of study participants or cases of vaccine breakthrough have been, and continue to often be, caused by SARS-CoV-2 VOCs that escape the immune responses induced during primary immunisation (e.g (70).). Although many studies sequence the virus from reinfected participants to confirm infection by a VOC, this complicates the interpretation of immunological parameters associated with protection. Specifically, it is difficult to extrapolate whether prior immunisation provided, or would have provided, strain-specific protection versus broad-range protection.

As the timing of viral challenge is also unknown, the viral and immune trajectories at critical timepoints post-challenge cannot be studied in human trials. Strong correlations between the IFI27 biomarker and a subset of memory CD8+ T cells, implied HCoV cross-reactive SARS-CoV-2-reactive memory CD8+ T cells to facilitate ‘abortive SARS-CoV-2 infection’ (59). However, if reinfection were to have occurred in a controlled manner, many immune parameters could have been traced. This would rule out the possibility that this CD8+ T cell subset is only a consequential biomarker of controlled infection, with a different mechanism actually being responsible for viral control.

Only controlled human challenge studies can overcome the aforementioned limitations of human clinical trials and longitudinal studies. However, human challenge studies are associated with high cost and risk, and in the case of SARS-CoV-2, there is also a paucity of naïve participants due to vaccination or previous challenge (71)). Therefore, though extremely valuable, human challenge studies are infrequently performed. Animal challenge studies not only fill this void, but also overcome the drawbacks of human challenge trials.

Considering the many limitations of COVID-19 vaccine efficacy clinical trials discussed above, there are several obvious benefits of animal models. Animal models overcome biases, lack of attendance to screenings and escape of asymptomatic cases, as frequent mandatory testing is considerably more feasible. In pre-clinical vaccine or rechallenge studies, animals are intranasally- and intratracheally-challenged with matched inoculation doses following vaccination. In which case there is a known, comparable exposure event, and the endpoint of protection can be determined with near certainty. Animal studies also follow identical regimens for vaccine dose administration, challenge and rechallenge, therefore immunologists can map immune landscapes at each critical timepoint. Carrying out challenge and testing under the same, known conditions facilitates direct comparability and the extraction of CoPs with greater confidence.

It is also important to remember that the definition of CoPs relies on a range of outcomes arising post-immunisation to extract immune profiles that differentiate protected from unprotected groups. However, stratification of outcomes was limited in human trials as, a) suboptimal SARS-CoV-2 vaccines did not progress through human clinical trials, b) the inclusion of placebo control groups in SARS-CoV-2 vaccine trials during the pandemic was an ethical dilemma (72) which limited the duration of study of these groups, and c) SARS-CoV-2 vaccine candidate efficacy was high. Pre-clinical studies however involved the testing of a range of vaccines with different efficacies, different dose numbers, dosing intervals and formulations, under the same conditions to yield an array of phenotypes post-challenge that increases the power behind the correlations that are drawn between immune parameters and protection.

Another advantage of using animal models to define CoPs, is that research animals, often contained within closed facilities, have a more definable immunological history in comparison to human participants that have a more diverse and undetermined virome and bacteriome. This is particularly relevant as human studies have identified a CD8+ T cell response against SARS-CoV-2 Nucleocapsid (N_105-113_) epitope, presented by HLA-B*07:02, in 80% of naïve participants and is significantly associated with mild COVID-19 (73–76). This response has been proposed to originate from a seasonal human coronavirus (HCoV) infection, due to the marked homology between this SARS-CoV-2 Nucleocapsid epitope and HCoV-OC43 and HCoV-HKU1 Nucleocapsid (73–76). Similarly, Swadling et al. proposed that pre-existing memory T cells against the highly conserved HCoV proteins NSP7, NSP12, and NSP13 of the replication transcription complex (RTC) facilitates abortive infection in seronegative HCWs (59), as also reported by Kundu et al. (77). Upon a complex immunological background, it is difficult to conclude the source of protection. Animal challenge studies however, can describe more accurately the specific immune signature left by SARS-CoV-2 vaccination and infection, as they are less likely to have been exposed to closely-related HCoVs.

**Figure 2:**
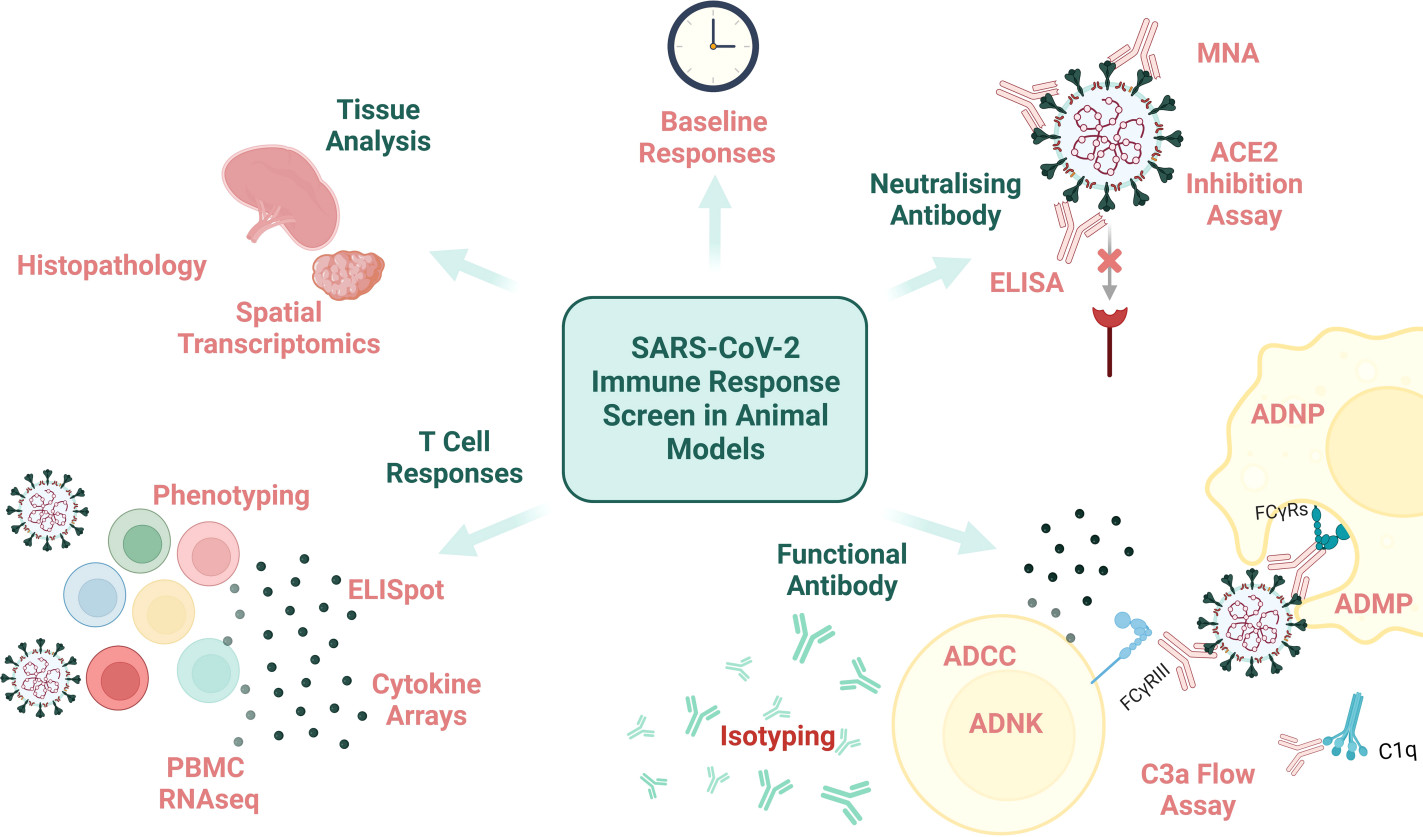
An overview of immunological assays and analysis that can be performed on animal samples. MNA, microneutralisation assay; ELISA, enzyme-linked immunosorbent assay; ADNP, antibody-dependent neutrophil phagocytosis; ADMP, antibody-dependent macrophage phagocytosis; FcyRs, fragment crystallisable of antibody receptor; ADCC, antibody-dependent cellular cytotoxicity; ADNK, antibody-dependent NK cell activation; PBMC, peripheral blood mononuclear cells; RNAseq, RNA sequencing. Created with BioRender.com.

An array of clinical (CT, X-ray), immunological (serology and blood analysis) and viral (PCR from URT and lower respiratory tract (LRT)) assessments can be carried out on human challenge study participants at timepoints throughout immunisation and acute infection to capture the clinical, immune and viral dissemination trajectories. However, the thoroughness of pathological assessment of a human subject will never match that possible in animal subjects (except perhaps in the context of post-mortem analysis of human COVID-19 fatalities (78), which is not as relevant for CoP identification). Performing histopathologic assessment of animal tissue post-cull supports the precise definition and stratification of disease endpoints which can be compiled with immunological data to reach conclusions on CoPs for the disease-of-interest (examples of measurable immunological data are demonstrated in **Figure 2**).

**Figure 3:**
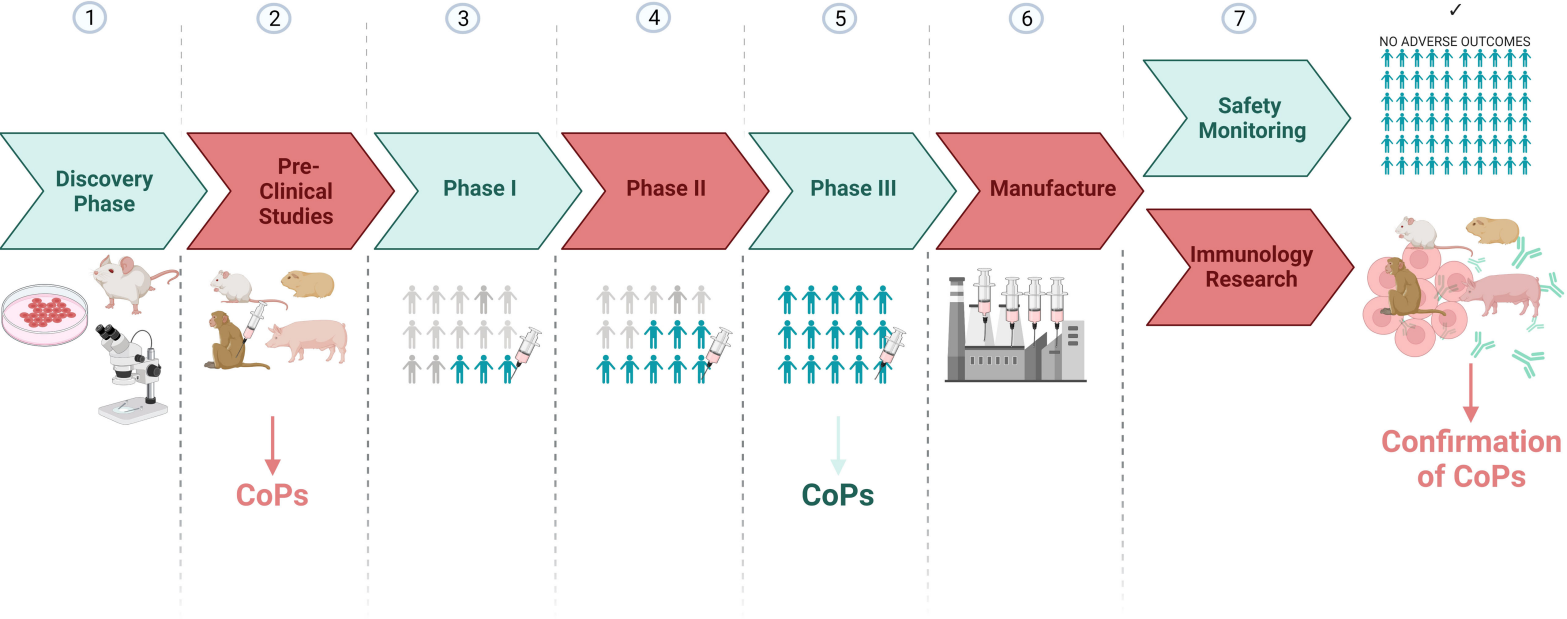
A vaccine development pipeline highlighting the stages where candidate CoPs can be identified and confirmed. Created with BioRender.com.

Candidate CoPs identified from preclinical and/or human phase III vaccine trials, can also be validated in later immunology research studies involving animal models (**Figure 3**). For example, animal thymectomies, cell depletion or adoptive transfer experiments can be carried out to determine whether the feature identified in human phase III studies directly explains protection outcomes when an animal is rechallenged with SARS-CoV-2. Hence outputs from clinical research can also be complemented by animal research.

## 5 Strategies to facilitate CoP identification in COVID-19 animal models

### 5.1 Adoptive transfer and cell depleted animal models

**Figure 4:**
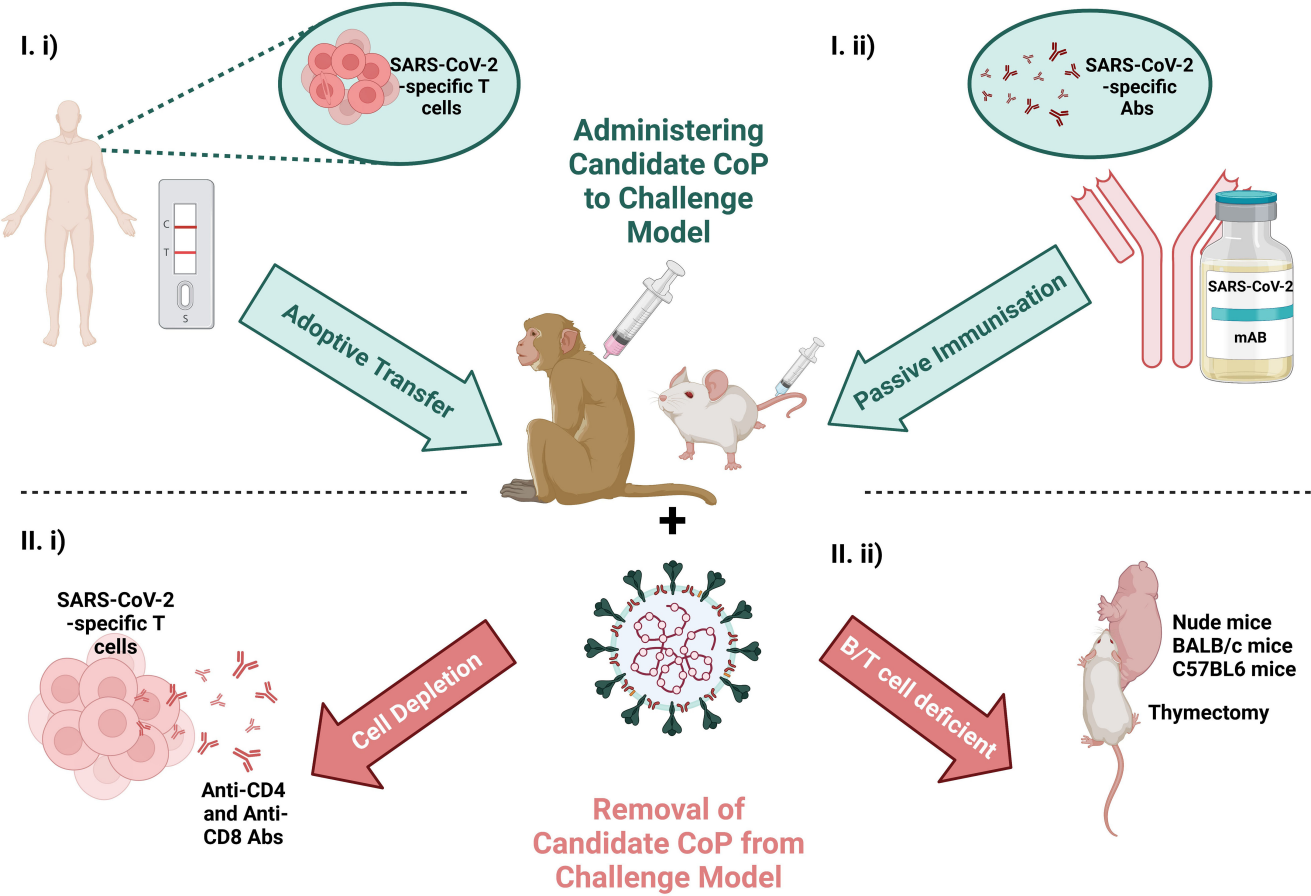
Schematic of the scope to manipulate the immunological response by I) administering the candidate CoP to the challenge model (at top in blue) *via* i) adoptive transfer of T cells from a convalescent individual or ii) passive immunisation with convalescent serum or a therapeutic mAb or II), removing candidate CoP from challenge model (at bottom in red) *via* i) T cell depletion or ii) use of an immunodeficient animal model. Challenging the animal with virus and ascertaining the effects of removal/addition of these immune features on virology and pathology, can provide evidence for/against the candidate CoPs. Created with BioRender.com.

As mentioned, one of the major advantages of using animal models for the identification of CoPs is the capacity to manipulate the immune response post-immunisation to demonstrate the consequence of introducing or withdrawing immune parameters on outcome post-challenge (described in **Figure 4**).

The independent role of T cells in protection against SARS-CoV-2, was investigated by McMahan et al. and Hasenkrug et al. in the macaque challenge model for SARS-CoV-2 by performing T cell depletion experiments.

Hasenkrug et al.’s experiment involved anti-CD4 and anti-CD8a monoclonal antibody (mAb)-mediated depletion of CD4+ and CD8+ T cells, prior to primary challenge with ~4 x 10^5^ TCID_50_. In the four CD8+ T cell-depleted RhMs, they report amplified CD4+ T cell numbers and responsivity in the cervical lymph nodes (LNs) and spleen during acute infection, likely to compensate for the lack of CD8+ T cell-mediated cellular adaptive immunity. Viral load measurements by qPCR confirms infection clearance in the control group by day fourteen, while in T cell-depleted groups, the infection was resolved by day twenty-one. Therefore, while the delay in viral clearance is likely attributable to the lack of T cell response, eventual clearance is not impacted by impaired T cell activity (28).

In contrast to Hasenkrug et al. that depleted the T cell subsets prior to primary infection, McMahan et al. depleted CD8+ T cells seven weeks post-primary infection, prior to rechallenge with 1 x 10^5^ TCID_50_, to investigate the role of memory CD8+ T cells in protection against reinfection. In comparison to the five control sham-mAb treated RhMs, that were successfully protected from reinfection, 100% and 25% of the eight CD8-depleted RhMs had detectable SARS-CoV-2 RNA in nasal swabs and BAL samples, respectively (10). Together, the results of these RhMs studies, implies a role for T cells in controlling viral load.

Despite expansion of receptor-binding domain (RBD)-responsive memory CD4+ and CD8+ T cells post-vaccination of hACE2-C57BL/6 mice with an alum-adjuvanted recombinant RBD vaccine, adoptive transfer of splenic CD4+ and CD8+ T cells from vaccinated-mice did not protect recipient naïve mice upon challenge, but passive transfer of immunised sera was found to be protective (9). Similarly, Matchett et al. reports on the persistence and expansion of Nucleocapsid (N_219-227_)-specific memory CD8+ T cells in the lung and lung draining mediastinal LN post-challenge and post-successful vaccination with a SARS-CoV-2 Nucleocapsid-expressing human adenoviral vector 5 (HAd5)-vectored vaccine. However CD8+ T cell depletion only partially abrogated protection upon challenge of vaccinated K18-ACE2 C57BL/6 mice (40). While these results are most likely due to the mouse less faithfully modelling the human immune system, it is also possible that T cells are only a ‘surrogate’ of protection, that support the emergence of a protective humoral response.

Understanding the relationship between the cellular and humoral response in the context of COVID-19 can also be thoroughly investigated *via* T cell manipulation experiments in animal models. The aforementioned study by Hasenkug et al., observed significantly attenuated peripheral B cell responses in the CD4+ T cell-depleted RhMs in comparison to control animals, in all but the animal that failed to achieve >90% CD4-depletion, confirming the involvement of CD4+ T cells at this adaptive axis (28). A delayed induction of IgM or isotype switching post-challenge was also observed in 50% of CD4+ T cell-depleted RhMs (28). This rate of dependency on CD4+ T cells for the induction of an antibody response explains the positive correlation between anti-Spike or RBD IgG titres and Spike-specific CD4+ T cell frequency and activity, found in human studies (79). However, the effect of CD4+ T cell-depletion on antibody development had no additional consequence on outcome post-challenge of this RhM cohort (28). Therefore CD8+ T cells may sufficiently mediate protection under these circumstances, as implied by the findings of McMahan et al.

Rydyznski et al. reports that the three arms of the antigen-specific adaptive immune response (antibody, CD4+ and CD8+ T cells) were mounted successfully in 73% of mild human COVID-19 cases, with unsuccessful coordination of such responses occuring in the elderly (greater than sixty-five years old) most prone to severe COVID-19 disease (80). The RhM SARS-CoV-2 model confirms a role for T cells in protection, with evidence thus far suggestive of T cell responses supporting viral control. Further work is necessary to underpin the true weight of the role of T cells in providing protection and to define the precise T cell population responsible for protection.

### 5.2 Passive transfer of antibody to animal models

The scope to manipulate animal immune responses to identify the parameters crucial for protection continues with passive transfer of antibody. Given the above indications that the role of T cells impacts B cells responsivity and antibody titres, proof-of-concept that antibodies are in fact the key mediators of infection resolution and protection can be achieved *via* the passive transfer of antibody to animals involved in pre-clinical vaccine and rechallenge studies.

Rogers et al. isolated S+ and RBD+ memory B cells (MBCs) from eight SARS-CoV-2 human convalescent donors and neutralising mAbs were passively transferred to Syrian hamsters by intraperitoneal infusion at five different concentrations. Twelve hours later, the animals were intranasally challenged with a dose of 1 x 10^6^ PFU of SARS-CoV-2. Using weight loss as the measure of disease magnitude, neutralising antibody (NAb) titres of ~22 ug/ml and 12 ug/ml, confers full protection or a 50% reduction in disease burden, respectively (35). Similarly, passive transfer of 10 mg of mRNA-1273-vaccinated RhM IgG to Syrian Hamsters also provided protection upon SARS-CoV-2 challenge (but 2 mg did not) (81).

McMahan et al. isolated SARS-CoV-2-specific NAbs from nine challenged macaques. Twelve RhMs, divided into four groups, were intravenously infused with concentrations of IgG that differed by an order of magnitude and were subsequently challenged with 1 x 10^5^ TCID_50_ of SARS-CoV-2. A dose-dependent effect of SARS-CoV-2-specific NAb titrations on viral load was observed, with the group infused with the highest IgG titres (250 mg/kg) yielding negative PCR results from BAL and nasal swab samples and hence are protected from infection. McMahan et al. was the first to propose NAbs as a CoP for COVID-19, as it is an immune parameter that significantly differentiates protected from non-protected NHPs and correlates with protection (10).

The pre-clinical evaluation of pharmaceutical mAbs for their therapeutic and/or prophylactic effects, represents another setting for experimental passive transfer of NAbs to animals, to assess the role of antibody in protection against SARS-CoV-2 challenge. Two mAbs, tixagevimab and cilgavimab, of AstraZeneca’s Evusheld, which potently and collaboratively target the ‘open’ and ‘closed’ conformations of the ACE2 RBD, reduce pathology and viral load when tested as a therapeutic intervention and provides protection when administered prophylactically to female hACE2-BALB/c mice (44). Similarly, the REGN-COV pre-clinical trial of the mAb cocktail, casirivamab plus imdevimab, successfully limited pathology and viral load when administered prophylactically and therapeutically in the mild COVID-19 RhM challenge model and severe COVID-19 Syrian golden hamster challenge model with low and high dose SARS-CoV-2 inocula (12).

The functional capacity of the Fc domain of passively transferred antibody must also be considered. McMahan et al. found functional antibody responses including antibody-dependent complement deposition (ADCD), antibody-dependent NK cell activation (ADNKA) and antibody-dependent neutrophil phagocytosis (ADNP) to correlate with protection in their passive transfer experiments (10). Administering genetically-engineered antibody to animals has also accelerated the field’s understanding of the role of the Fc domain of antibody in the context of SARS-CoV-2 infection. An Fc-mutated mAb fails to confer clinical, viral and pathological protection when administered therapeutically to both K18-hACE2 transgenic mice and Syrian hamsters, however the functional Fc mAb did successfully protect these animal models in the early days post-challenge (13). This is suggestive of a crucial role for the Fc domain in the control of acute infection in these models. Whether the Fc has a prophylactic role is less clear. Serum levels of intraperitoneally administered anti-RBD NAb, prior to intranasal challenge with 1 x 10^3^ PFU of SARS-CoV-2, correlated with clinical protection from COVID-19 and inversely correlated with lung vRNA in the K18-hACE2 transgenic mouse model, irrespective of whether the Fc region was loss-of-function mutated (13). Meanwhile, a non-RBD-based S2 stem helix-targeting neutralising mAb, S2P6, that activates Fc-mediated effector functions antibody-dependent cellular cytotoxicity (ADCC) and antibody-dependent cellular phagocytosis (ADCP), effectively limits lung vRNA when administered prophylactically in the Syrian hamster challenge model (36). These results demonstrate the potential collaborative effect of neutralising and Fc functional SARS-CoV-2-specific antibodies to confer complete protection.

Additional immune manipulation strategies facilitated the interrogation of the potential mechanisms underlying antibody Fc-mediated protection. The investigative strategies adopted were based on the observation that functional Fc mAb-treated mice had reduced counts of TNFa+iNOS+CD80+CD11b+ monocytes and an amplified frequency of activated CD8+ T cells (13). Depletion of monocytes in functional Fc mAb-treated mice, resulted in the loss of clinical protection, yet a sustained ability to reduce viral burden. Depletion of CD8+ T cells in functional Fc mAb-treated mice contributed to the loss of viral control, but not a loss of clinical protection (13). This is in support of the aforementioned T cell depletion experiments that proposed T cells as mediators of viral control. The proposed mechanism of protection of functional Fc mAb-treated mice is that phagocytosis and antigen presentation follows virus-Fab-Fc-FcyR immune complex formation on monocytes, so as SARS-CoV-2-reactive cytotoxic CD8+ T cells can be activated and destroy virally infected cells (13).

### 5.3 High versus low dose vaccine comparison

**Figure 5:**
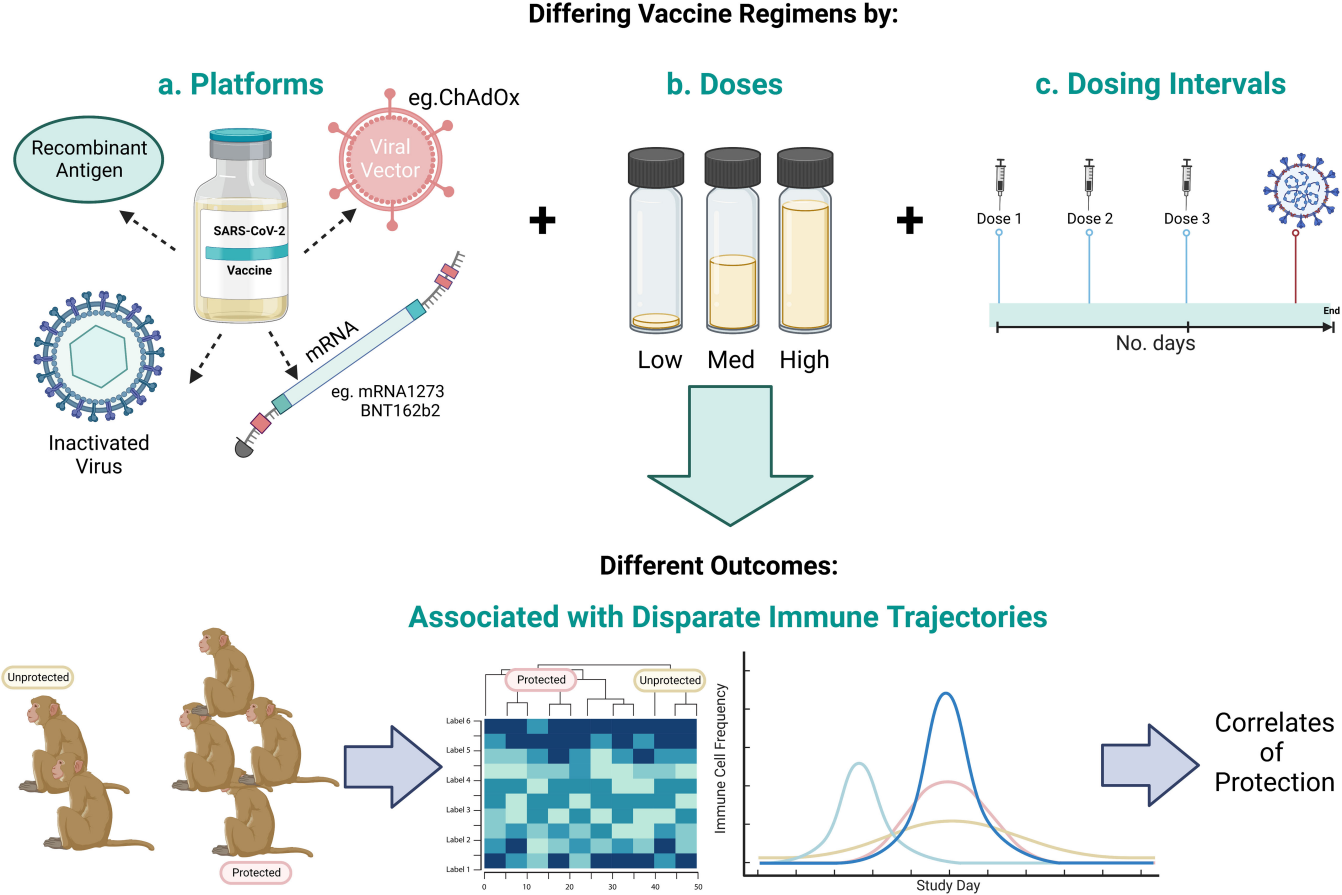
Schematic representing the scope to identify CoPs by investigating a range of a) vaccine candidate platforms, b) number of vaccine doses, and c) vaccine dosing regimens to give a range of outcomes in order to identify the immune profile that differentiates protected from unprotected animals. Created with BioRender.

The identification of a CoP relies equally on vaccine breakthrough as it does successful immunisation in order to stratify outcomes and define the immune profiles that differentiate protected from unprotected groups. Formulated in lipid nanoparticles (LNPs), Moderna’s mRNA vaccine, mRNA-1273, was 94.1% effective at preventing symptomatic disease and 100% effective in preventing severe disease, when two doses were administered twenty-eight days apart in a phase III trial. However, given the efficacy of the vaccine, with only eleven of 15,210 vaccinated participants (0.07%) contracting COVID-19, CoPs could not be identified (67). As in the name, CoP, statistical power and ‘correlations’ underpin the investigation of candidate CoPs, therefore the low number of vaccine breakthrough cases means this need will not be met by human SARS-CoV-2 vaccine clinical trial data. Hence, pre-clinical vaccine studies that investigate protective and sub-protective vaccine dosing strategies under matched conditions, diversifies challenge outcomes and immune profiles, from which statistically significant correlations with protection can be drawn (summarised in **Figure 5**).

BALB/cJ, C57BL/6J and B6C3F1/J mice were intramuscularly vaccinated with mRNA-1273 as part of a two-dose regimen with three week intervals (47), as were twelve RhMs at four week intervals (14, 47). A vaccine dose-dependent effect on binding and NAb emergence was observed (14). Challenge of vaccinated BALB/cJ mice with 1 x 10^5^ PFU of SARS-CoV-2 at week five or week thirteen post-boost, and vaccinated RhMs challenged with 7.6 x 10^5^ PFU at week four post-boost, revealed a vaccine dose-dependent reduction in lung viral load (47), such that NAb responses were negatively correlated with viral load in the nasal turbinates (14).

He et al. found that the lowest dose of Ad26.COV2.S kept SARS-CoV-2 sgRNA levels at a minimum in the LRT of RhMs challenged with 1 x 10^5^ TCID_50_ six weeks post-single-dose vaccination. However, a higher vaccine dose also protected against the establishment of a SARS-CoV-2 infection in the URT, in addition to the LRT (15). This was attributed to the trend for poorer anti-RBD IgG and NAb kinetics and response magnitudes, as well as reduced T cell and RBD-specific IgG+ MBC activity in the low dose vaccine groups (15). Specifically, the MBC compartment was amplified in the higher dose groups, which was found to be associated with completely protected groups, in comparison to non-protected or partially protected groups. MBC frequency positively correlated with respective antibody titres and negatively correlated with nasal swab sgRNA levels (15).

Minimal lung pathology and a significant decline in viral load was observed in RhMs vaccinated with a high dose of beta-propiolactone-inactivated SARS-CoV-2 vaccine candidate, PicoVacc. This was observed following intratracheal challenge (1 x 10^6^ TCID_50_) one week post-completion of the three-dose regimen (16). Medium-dose vaccinated animals induced lower NAb titres and increased incidence of SARS-CoV-2 detection in the pharynx and lung. These results were replicated in PiCoVacc-immunised BALB/c mice and Wistar rats, providing additional support for NAb as a mediator of viral control (16). No significant difference in CD3+, CD4+ or CD8+ frequency, or inflammatory cytokines, were noted between the vaccinated and control groups (16).

From the above-mentioned vaccine pre-clinical studies, a vaccine dose-dependent effect on antibody titres was observed, that subsequently correlated with viral load. This research highlights the necessity for a range of vaccine doses to be tested in animals in order to draw associations between immune profiles and protection.

### 5.4 Comparison of matched optimal and sub-optimal vaccine candidates

Many vaccine platforms, such as vector-based vaccines or nucleic acid vaccines, are amenable to the testing of different antigenic components that may differ in their immunogenicity and hence in the level of protection the induced immune response provides. Hence, pre-clinical studies of vaccines, that differ only in their antigen composition, provides another mechanism of promoting challenge outcome and immune profile divergence that favours SARS-CoV-2 CoP identification.

Ad26 vector-based vaccines, incorporating different forms of SARS-CoV-2 Spike (Spike sequences that differ in length, that incorporate the furin cleavage site mutation and/or further stabilising mutations) were first tested in RhMs (17). Due to the range of challenge outcomes yielded post-vaccination with the different Ad26 candidate vaccines, the group concluded that NAb titres were the factor that differentiated protected from unprotected RhMs post-challenge, with ADNKA and ADCP also contributing to the separation of these protection statuses (17). In fact, it was proposed that the collaborative effect of antibody neutralisation and Fc-mediated effector functions had an improved correlation with protection (revealed following logistic regression analyses) (17), supportive of the results from the therapeutic Fc functional mAb pre-clinical trial discussed above (13).

The Ad26 vaccine encoding pre-fusion stabilised full-length Spike (Ad26.COV2.S) generated the most substantial immunological effector functions and viral control responses, with BAL samples from RhMs in this group lacking detectable virus upon challenge with 1 x 10^5^ TCID_50_ of SARS-CoV-2 at week six (17). This evidence-based optimal vaccine was also tested against a suboptimal Ad26 SARS-CoV-2 vaccine in fifty Syrian golden hamsters that model more severe COVID-19. An array of outcomes post-intranasal challenge with 5 x 10^5^ TCID_50_ of SARS-CoV-2 were elicited, including successful, partial and failed protection which support CoP identification. Clinical and viral outcomes were also found to inversely correlate with anti-RBD and/or NAb responses in this model (32).

Formulated in LNPs, Pfizer/BioNTech’s mRNA vaccine candidates, BNT162b1 and BNT162b2, encoded soluble RBD or pre-fusion stabilised full-length Spike, respectively (18). The intranasal and intratracheal challenge of twelve BNT162b1/BNT162b2-immunised and nine control RhMs with 1.05 x 10^6^ PFU of SARS-CoV-2, revealed that BNT162b2-vaccinated macaques’ BAL PCR results remained negative throughout post-challenge sampling, in comparison to the control and BNT162b1-vaccinated macaques that had a higher incidence of BAL PCR vRNA positivity (18). At challenge, matched neutralising responses were seen in RhMs vaccinated with the BNT162 candidates, hence the aetiology of the improved BNT162b2 efficacy does not fall with enhanced neutralising responses. Augmented levels of circulating CD8+ T cells were detected in mice vaccinated with BNT162b2 versus BNT162b1, likely due to the broader range of T cell epitopes encoded by the BNT162b2 candidate. However, mice were not challenged as part of this study to relate this disparity to protection outcomes (18).

Thirty-five RhMs were intramuscularly vaccinated with one of six DNA vaccine candidates, each encoding different SARS-CoV-2 Spike variants, or the sham vaccine, to induce heterogenous response profiles (19). Subsequent challenge with 1.1 x 10^4^ PFU of SARS-CoV-2 *via* intranasal and intratracheal routes, resulted in a ~two-fold reduction in median BAL and nasal viral loads in vaccinated groups in comparison to the sham control group (19). URT and LRT sgRNA levels were found to inversely correlate with NAb titres (with a systems biology approach indicating a potential collaborative effect with Fc-mediated responses of ADCD and ADCP), while ELISpot and ICS results did not correlate, inferring that the humoral and not the cellular compartment mediates this protection (19).

Efficacy of CureVac’s LNP-formulated SARS-CoV-2 mRNA vaccine, CVnCoV, was observed in twenty Syrian golden hamsters in the form of reduced lung pathology, minimal URT and undetectable LRT viral loads (37). RhMs that received high dose CVnCoV vaccination also experienced significantly reduced lung lesion severity and undetectable LRT vRNA (20). However a statistically significant difference in URT vRNA copies between high dose CVnCoV recipient versus unvaccinated/low dose CVnCoV-vaccinated RhMs was not observed following intranasal and intratracheal challenge with 5 x 10^6^ PFU (20), which may provide an explanation for the poor 48.2% efficacy reported from clinical trials, irrespective of the induction of Spike- and RBD-specific IgG and NAbs (82). The failure of the first generation CureVac vaccine was also attributed to the incidence rate of breakthrough infections caused by VOCs (82).

Hence, a second generation CureVac SARS-CoV-2 vaccine (CV2CoV) was developed with enhanced intracellular Spike transcript stability to optimise antigen expression (83). Following positive initial immunogenicity and efficacy results of a high dose prime-boost CV2CoV regimen in Wistar rats (83, 84), a comparison of CVnCoV and CV2CoV vaccines was drawn in eighteen CyMs, inclusive of six control CyMs, which yielded a range of immune profiles and outcomes (30). The higher innate cell, NAb, MBC and T cell responses post-CV2CoV vaccination versus CVnCoV vaccination, coincided with lower sgRNA copies in the URT and LRT post-intranasal and -intratracheal challenge with 1 x 10^5^ TCID_50_ eight weeks post-vaccination with CV2CoV (30). In fact, NAb titres at two weeks post-boost were found to inversely correlate with BAL and nasal swab sgRNA (30). This is another example of how side-by-side comparisons of vaccines accelerate CoP identification.

Direct comparison of sub-optimal and optimal vaccines, mediates the partitioning of outcomes and immune profiles to identify correlations between immune parameters and protection. A vaccine developed to interrogate the vaccine-associated enhanced disease (VAED)-potential of SARS-CoV-2 vaccines, a formaldehyde-inactivated viral (FIV) SARS-CoV-2 vaccine with a Th2-skewing adjuvant, alhydrogel, was studied in ferrets and RhMs and further supports SARS-CoV-2 CoP investigations (21). While a single dose of FIV did not provide clinical protection (there were insignificant differences between FIV-vaccinated and sham control RhM CT scores, weights and temperatures), vaccinated RhMs did yield significantly lower mean vRNA concentrations, pathology scores and infected lung area post-intranasal and -intratracheal challenge with 5 x 10^6^ PFU of SARS-CoV-2. Deeper analysis of the immune profile associated with this ‘sub-optimal’ protection, revealed that FIV vaccination only elicited a modest neutralising response in ferrets and RhMs providing the most likely explanation for the lack of protective efficacy (21). This example demonstrates how sub-optimal vaccination highlights deficiencies in the immune response, not seen in optimally-vaccinated animals, that ultimately contribute to the lack of protection, thus increasing our confidence in candidate CoPs.

### 5.5 One versus two vaccine dose comparison

Pre-clinical efficacy studies often investigate the number of vaccine doses required to achieve optimal protection. As a result of this exploratory research, control unvaccinated, primed-only and prime-boosted groups of animals yield a hierarchy of outcomes, from which parameters that distinguish protected from unprotected animals can be identified to inform CoP research. This approach offers significant advantages as monitoring the protective efficacy post-prime, and then subsequently post-boost, in the same animal, can illustrate the trajectory of protective immune response development which may help to identify predictors of immunity. Additionally, it must be considered that protection may be provided *via* one mechanism post-prime, that evolves to establish a different protective profile post-boost.

The preclinical study of Ad26.COV2.S involving sixty RhMs, found a two-dose regimen to fail to improve protective efficacy as the median number of days with detectable sgRNA in the URT was minimally different between the primed-only and prime-boosted NHPs, despite significantly higher anti-Spike IgG and NAb responses in boosted-RhMs (22). While the boost similarly had a 2.6-2.9-fold amplification effect on NAb titres of Ad26.COV2.S-vaccinated humans, this did not improve protective efficacy, and provides the rationale for Janssen’s adoption of a single-dose regimen (85).

Though the Ad26.COV2.S studies illustrated that one dose was optimal, many of the approved SARS-CoV-2 vaccines adopted a two-dose regimen. Whilst two doses were found to improve the immunogenicity, response longevity and efficacy of these vaccines, many did provide considerable protection post-prime also. In which case, what vaccine-induced response/s is/are responsible for the primary protection and the enhanced protection achieved post-boost?

In the multicontinental clinical trial of ChAdOx-1 nCoV-19, the vaccine was 64.1% protective post-prime, not-too-dissimilar to the 70.4% efficacy reported post-boost (86). NAb titres over the twenty-seven days post-prime of RhMs with ChAdOx-1 nCoV-19 were found to increase (23, 24). This is concordant with the natural increase in NAb titres and the frequency of responders eliciting a neutralising response from week four post-Ad26.COV2.S prime which provides sufficient protection (85).

An evolving humoral profile during the interval between prime and boost is also observed with Pfizer/BioNTech’s SARS-CoV-2 vaccine, BNT162b2. Thomas et al. reported an increase in protective efficacy from 58.4% post-prime to 91.7% from day eleven post-prime to day twenty-one (the day of boost), in 43,409 human participants vaccinated with BNT162b2 (87). The antibody profile post-BNT162b2 prime, reported by Walsh et al., was predominantly non-neutralising, even in the 50% of vaccine recipients that had detectable neutralising responses (88). Protection may therefore be explained by the evolution of non-neutralising antibody with enhanced Fc-effector functionality post-prime (88, 89). In which case, antibody ‘quality’ rather than ‘quantity’ may be responsible for BNT162b2-mediated protection. Alternatively, these results may be explained by *in vitro* neutralisation assay limitations, such as sensitivity (90), and disregard for the contribution of other serum factors, such as complement, in neutralisation, as reported by Mellors et al. for Ebola virus (91).

Therefore, the boost-induced superior protective response is explained by which immune parameters? NAb titres do increase in humans post-boost with ChAdOx-1 nCoV-19, which correlate with viral and clinical protection and likely contributes to the improved efficacy of ChAdOx-1 nCoV-19 to 70.4% post-boost (23, 38). However, an increase in IgG1 and IgG3 titres may also contribute to this improvement (92). A second dose of BNT162b2, which is reported to improve vaccine efficacy supports the emergence of potent and broadly-neutralising antibody and a predominately class-switched IgG+ SARS-CoV-2-specific MBC repertoire in humans (88, 89).

However, in pre-clinical studies of Novavax’s vaccine, NVX-CoV2373, largely equivalent NAb titres are induced in the single and double-dosed groups. Multivariate analysis revealed that multi-subclass Spike-specific Ig responses, ADCD and NAbs separate fully protected RhMs (both URT and LRT protection) from partially (LRT protection only) or unprotected RhMs. As partially or unprotected RhMs had a poorer ability to drive Fc-mediated effector functions, and functional antibodies explosively mature post-boost with respect to the less dramatic change in NAb titres post-boost, functional antibody may underpin the enhanced efficacy of a two-dose regimen of NVX-CoV2373 (27). In the phase III NVX-CoV2373 trial involving 14,039 participants that took place during Alpha variant circulation, eight of ten vaccine breakthrough cases were Alpha variant infections (70). This observation can be explained by the discovery that RhM and human antibodies lack the ability to simultaneously bind both the FcR and SARS-CoV-2 variants that harbour the E484K mutation, such as the Alpha variant (27). This real-world scenario provided additional support for the role of the Fc of NVX-CoV2373-induced SARS-CoV-2-specific antibody in mediating protection.

A combination of data collated from animal and human trials has aided understanding of the evolution of the immune response required for optimal vaccine-mediated protection. The combination of animal and human efficacy and immunogenicity data post-prime and -boost has been used to deduce that functional binding antibody and NAbs are strong CoP candidates.

### 5.6 The use of different adjuvants

Optimising a vaccine’s adjuvanticity is a crucial consideration in any vaccine design process. Adjuvanticity describes a vaccine’s ability to stimulate innate immune cells (required for the eventual induction of an antigen-specific response by adaptive immune cells) mediated by the ‘adjuvant’ component of the vaccine formulation. A number of factors influence a vaccinologist’s decision to use a particular adjuvant, including safety profiles, vaccine dose-sparing aims and a pathogen’s CoP, particularly were protection to be T cell subset-dependent (93). Commonly used adjuvants include water-in-oil emulsions, aluminium-containing adjuvants, pattern recognition receptors and LNPs (94). The use of the Th2-skewing alhydrogel adjuvant for the FIV SARS-CoV-2 vaccine discussed above, for example, highlights the capacity of adjuvants to diversify the post-challenge outcomes for the investigation of CoPs in pre-clinical vaccine studies.

Pre-clinical studies support the optimisation of vaccine immunogenicity *via* the testing of different adjuvants. For example, hACE2-BALB/c mice primed and boosted with NVX-CoV2373, of recombinant Spike plus saponin-based Matrix-M adjuvant, achieved higher frequencies of multifunctional effector memory T cells, T follicular helper (Tfh) cells and germinal centre (GC) B cells, as well as an amplified anti-Spike antibody response, than those administered the vaccine that lacked the Matrix-M adjuvant. This enhanced immunogenicity likely explains the minimal virology and pathology seen in SARS-CoV-2-challenged hACE2-BALB/c mice, CyMs and RhMs vaccinated with NVX-CoV2373, in comparison to groups that received the vaccine lacking the Matrix-M adjuvant (31, 45).

Many adjuvants have been investigated and compared under the same experimental conditions in animal models, in an attempt to optimise SARS-CoV-2 vaccine candidate efficacy. Arunchalam et al. reports that different adjuvants yield an array of COVID-19 outcomes. AS03-adjuvanted RBD-nanoparticle-vaccinated RhMs were found to be the most protected upon intranasal and intratracheal challenge with 3.2 x 10^6^ PFU of SARS-CoV-2 at week four, with undetectable vRNA in pharyngeal, nasal and BAL samples. AS03 adjuvant was found to induce the highest NAb titres, with NAbs significantly correlating with protection in this study. This NAb response positively correlated with the CD4+ T cell response, with a balanced Th1-Th2 response, as well as a higher frequency of circulating Tfh cells, being attributable to the adjuvant in use (25). ADNP also differentiated protected from unprotected RhMs following partial least-squares discriminant analysis and negatively correlates with viral load, thus providing further evidence for the role of functional antibody (25).

With adjuvant as the basis of comparison in the aforementioned studies, Lederer et al. investigated the effects of adjuvant on GC reactions in a mouse model. Given the theorised adjuvanticity of the LNP formulation of mRNA vaccines, the GC reactions of SARS-CoV-2 mRNA-vaccinated BALB/c mice were compared with those seen post-vaccination with the less-optimal recombinant RBD vaccine candidate adjuvanted with Addavax, a MF59-like adjuvant (rRBD-AddaVax) (95). In the mRNA-vaccinated mice, the frequency of SARS-CoV-2-specific GC B cells in the inguinal LN and the popliteal draining LN remains elevated at day twenty-eight post-vaccination, reminiscent of prolonged GC reactions (95). On the contrary, rRBD-Addavax-vaccinated mice lack evidence for GC reactions and unsurprisingly, NAbs do not emerge (95). Additionally, in stark contrast to the poor magnitude and kinetics of the IgG1-dominant response seen in rRBD-Addavax-vaccinated mice, Th1-polarisation of Tfh cells in the mRNA-vaccinated mice ensures IgG2a and IgG2b class switching in this group (95). Influencing GC reactions *via* the adoption of different adjuvants in animal models, sheds further light on the cellular and humoral profiles associated with protection outcomes.

### 5.7 Vaccine recipients with variable immune functionality

Next, we must explore the idea that vaccinees may have aged immune systems, conditions associated with immunodeficiency, or are being treated with immunosuppressive drugs. Immune features that are naturally compromised in vaccinated individuals, can indirectly provide evidence for protective mechanisms. For example, failed protection in participants with immunoglobulin deficiency would provide support for the role of the humoral response in protection. Additionally, the potential for redundancy mechanisms to be at play in these recipients may also point to a ‘surrogate of protection’. This is particularly relevant due to the age-bias of COVID-19. The phenomena of ‘inflammageing’ and thymic involution in the aging population equally heightens the requirement for immunisation of this population, as it does explain their increased risk of severe infection and the potential for a failed vaccination. Often in early phase I human clinical trials, only healthy participants below the age of fifty-five years are enrolled. Aged and/or immunocompromised individuals are only included in much later trials and studies, and so there is a considerable lag before it is possible to investigate the immune response to vaccination in these populations.

Hence, ‘aged’ or immunocompromised animal models, can accelerate and further support this research. For example, lower antibody titres were reported in the aged Syrian golden hamster model with respect to the younger cohort. This difference in humoral response magnitude impacted their ability to protect against challenge with 1 x 10^5^ PFU of SARS-CoV-2 (33). While young hamsters had undetectable vRNA in the lung by day five and recovered from infection by day fourteen, aged hamsters had sustained high viral loads in the lung and persistent inflammation (33).

Silva-Cayetano et al. compared the immunogenicity of ChAdOx-1 nCoV-19 in three-month-old versus ‘aged’ twenty-two-month-old C57BL/6 mice. In the twenty-two-month-old ‘aged’ mouse model, the percentage of GC B cells post-ChAdOx-1 nCoV-19 vaccination was lower, coinciding with the absence of GCs in the spleen, reduced numbers of proliferating Tfh cells, an impaired type I IFN response, as well as lower anti-Spike IgG and NAb titres, as seen in older humans (46). The compromised GC response was rescued by the second ChAdOx-1 nCoV-19 vaccine dose in the aged mouse model, with draining LN plasma cells, GC B cells and Tfh cells being detectable by day nine post-boost, which occurs in parallel with an eight-fold increase in anti-Spike IgG and NAb responses (46). Similarly, human ChAdOx-1 nCoV-19 vaccine recipients over the age of seventy had lower Th1 cell frequencies post-prime, but both Spike-specific CD4+ and CD8+ T cell responses were elevated post-boost to match frequencies seen in the younger cohorts (96). Therefore, the vaccine was 61% effective between one to four weeks post-boost in recipients over the age of sixty-five (97). A boost also appears to be sufficient for the induction of a class-switched Spike-specific MBC response in immunosuppressed kidney transplant patients (98).

Recognising the immunogenicity and efficacy of different vaccination strategies for the more challenging vaccine recipient versus healthier vaccine recipients, can further enhance our understanding of candidate SARS-CoV-2 CoPs.

### 5.8 Alternative vaccine administration routes

The routes of entry of SARS-CoV-2, as a respiratory pathogen, include the mucosal sites of the respiratory system – the nose, throat and lung. Hence, to achieve ‘sterilising immunity,’ one may require sufficient SARS-CoV-2 reactivity at these sites. Therefore, induction of a strong SARS-CoV-2-specific mucosal immune response would likely improve vaccine efficacy, which intuitively can be achieved through intranasal or oral vaccination. The field of mucosal immunology has advanced over the last number of years (99). However rigorous research in animals is required prior to human trials of intranasal/oral vaccination, given the adverse events associated with this vaccine administration route, stemming from strong associations between an intranasally-administered influenza vaccine and the development of Bell’s Palsy in Switzerland (100). Therefore, animal models provide an opportunity to investigate mucosal vaccination, while also determining the role of mucosal responses in protection against a respiratory pathogen. Vaccination of animals *via* different administration routes will further diversify the immune response and challenge outcomes to deduce CoPs.

A comparison between the intramuscular (IM) and needle-free oral administration routes of an MVA-expressing Spike and Nucleocapsid vaccine, was addressed in RhM studies. Following challenge with 1 x 10^8^ PFU of the Delta variant four weeks post-boost, three protection outcomes were recorded; 1) robust protection *via* IM vaccination, 2) moderate protection *via* the buccal route, 3) failed protection *via* the sublingual route. A higher magnitude of serum and mucosal IgG, and functional Ab-dependent cellular activity was observed in the IM-vaccinated RhMs. Nasal anti-RBD IgG and NAbs, as well as serum ADCD, ADCP and ADNKA, were found to inversely correlate with viral load, providing further evidence for the collaborative efforts of neutralising and non-neutralising antibody to protect against SARS-CoV-2 challenge (101). T cell responses were comparable between the IM and buccal administration routes, hence they may contribute to protection also (101).

Adenoviruses are respiratory viruses, with binding affinity to the coxsackievirus and adenovirus receptor (CAR) expressed on respiratory mucosa, and are responsible for seasonal colds. Therefore unsurprisingly, intranasal (IN) administration of ChAdOx-1 nCoV-19 has been explored for its ability to induce lung-specific and systemic immune responses. ChAdOx-1 nCoV-19 IN administration has been shown to be associated with reduced pathology and URT and LRT virology post-challenge, when compared to IM administration in animal models (38, 49). A horizontal transmission experiment, whereby a naïve hamster is exposed to a challenged hamster for four hours (which more realistically mimics SARS-CoV-2 infection than direct intranasal inoculation), revealed that SARS-CoV-2 Nucleocapsid was undetectable in the lung tissue of the SARS-CoV-2-exposed IN-vaccinated hamsters, in comparison to control and IM-vaccinated hamsters where SARS-CoV-2 Nucleocapsid was detectable (39). This is reflective of LRT viral control, perhaps mediated by the six-fold higher titres of serum anti-Spike, anti-RBD and NAbs induced in IN- versus IM-vaccinated hamsters (38). However, whilst ChAdOx-1 nCoV-19 and Ad5-S-nb2 IN-vaccinated ferrets and RhMs were more protected than the IM-vaccinated animals following SARS-CoV-2 challenge, this cannot be explained by immune parameters that are measurable from blood samples (26, 49). In fact, IN-vaccinated animals failed to induce serum SARS-CoV-2-specific IgG titres or cell-mediated immune responses equivalent to those of IM-prime-boosted animals (26, 49).

It is possible and likely that mucosal rather than serum antibody is providing the improved protection observed following IN-vaccination. Upon challenge with 1 x 10^6^ TCID_50_ of SARS-CoV-2, IN-ChAdOx-1 nCoV-19-vaccinated RhMs had lower pathology, viral titre and frequency of virus detectability in the URT and LRT in comparison to controls (however many of these differences were insignificant) (39). Nasosorption sampling facilitated the analysis of the mucosal response to IN-vaccination, with mucosal SARS-CoV-2 IgA being detectable post-prime and amplified post-boost. A booster-effect on mucosal IgG was also observed from BAL sampling (39). Principle component analysis defined protected IN-vaccinated animals by their SARS-CoV-2-specific IgA and IgG responses in BAL and nasal samples, with correlations being drawn between nasal and BAL IgA and IgG samples and nasal and BAL vRNA, respectively (39). Similarly, IN-immunisation of female BALB/c mice with Ad5-S-nb2 induced anti-Spike IgA in the BAL, that was undetectable in the IM-immunised animals (26).

Mao et al. developed a vaccination strategy involving IM-priming with BNT162b2 mRNA vaccine, followed by an IN-boost with unadjuvanted recombinant prefusion-stabilised Spike, coined ‘Prime and Spike’. This vaccine strategy, administered to K18-hACE2 transgenic mice, elicited the amplification of nasal, lung and serum IgA and IgG, resident MBCs, long-lived plasma cells (LLPCs), and CD4+ and CD8+ tissue-resident memory (Trm) cells. In comparison to prime-only with a low dose of BNT162b2, known to be unprotective in the K18-hACE2 mouse model, this ‘Prime and Spike’ regimen significantly minimised lung pathology and reduced viral burden in the URT and LRT upon challenge with 6 x 10^4^ PFU. CD8+ Trm cells in the lung and BAL IgA were detected in the ‘Prime and Spike’ group only, while serum IgA and IgG, and BAL IgG, were matched between animals immunised *via* ‘Prime and Spike’ or prime-boosted with BNT162b2. As challenge was not performed, associations between these mucosal profiles and protection, could not be drawn, but informs the scope to induce mucosal immune responses by IN-vaccination (102).

In summary, comparison of vaccine administration routes in animal models, alludes to tissue-resident and mucosal immune features as CoPs for a respiratory pathogen such as SARS-CoV-2.

### 5.9 Tissue examination and manipulation scope

A major advantage of the use of animal models for CoP research is the scope for in-depth pathological analysis to better stratify post-challenge outcomes based on well-defined pathology scoring systems such as that seen in Salguero et al. (8). Additionally, an in-depth analysis of animal tissues such as lung, spleen and thymus, and the immune cell populations at these sites, can accelerate our search for a SARS-CoV-2 CoP.

Shaan Lakshmanappa et al. characterised the GC cell populations of RhMs by digesting LNs obtained at necropsy to generate a single cell suspension for flow cytometric analysis (11). A robust GC Tfh cell population in the mediastinal LN and spleen of RhMs was detectable following challenge with ~1.7 x 10^6^ TCID_50_ of SARS-CoV-2 intranasally, intratracheally and intraocularly (11). Bronchial-associated lymphoid tissue has also been observed in both RhMs and CyMs, with similar frequency and semblance, following pathological analysis, indicative of the induction of localised GC reactions upon challenge (8).

Lung and spleen isolated from BALB/c mice IM-vaccinated with an RBD, full-length Spike- or control luciferase-encoding LNP-formulated mRNA vaccine, revealed the emergence of polyfunctional IFNy+CD4+ and IFNy+CD8+ T cells in the spleen, and to a greater extent, in the lung parenchyma, demonstrative of lung homing and extravasation (103). At nine weeks post-vaccination, Spike- and RBD-specific IgG1+ and IgG2a/b+ MBCs in the spleen were detected, as were LLPCs of varying subsets in the bone marrow by flow cytometry and ELISpot analysis, revealing the scope for a durable protective response (103).

These studies capture the invaluable insights we gain from the in-depth analysis of animal tissue post-vaccination and post-challenge, that peripheral blood mononuclear cell (PBMC) samples from humans fail to provide. PBMC phenotypes are not demonstrative of Trms or GC cells in the LNs (98), hence we only capture a fraction of the immune cell landscape in the absence of tissue. Only study of human cadavers that succumbed to COVID-19 infection was carried out amidst the pandemic which highlighted the profiles associated with fatal COVID-19 (78), but could not aid CoP identification.

## 6 Future of CoP research in animals

### 6.1 Flaws of animal research in the search for CoPs

On the basis of the animal studies discussed above, a diverse response by the adaptive arm of the immune system is required for resolution and protection against SARS-CoV-2 infection. A downfall of the NHP challenge model for the definition of COVID-19 CoPs, is the high frequency of protection at reinfection (6), particularly frequent due to challenge with matched SARS-CoV-2 strains, the short intervals between vaccination and challenge, and the mild manifestation of this disease in the animals (except in old RhMs/CyMs). In which case, comparing immune parameters that differentiate protected from non-protected animals at rechallenge can be complicated unless precise pathology scoring systems are used.

Difficulties surrounding the breeding, handling and housing of the animals that most accurately recapitulate human COVID-19, i.e. RhMs and CyMs, contribute to the decision to cull animals soon after challenge. Such difficulties also limit the interval length between vaccine doses and between immunisation and challenge. While cull of animals soon after challenge/rechallenge captures the immune landscape during acute infection, this sacrifices the possibility for analysis of immune response durability and long-term immunity months post-immunisation, post-infection or post-reinfection.

Another clear limitation of animal models for the definition of a CoP, is the poor reproducibility of animal results in humans as was the case in the search for a rotavirus and HIV CoP using an NHP model (3). This is due to the lack of conservation of some immune features. For example, disparities between human and macaque NK cells include the high background activity of macaque NK cells (104) and the difference in frequency of cell surface marker expression (105, 106). This may explain why candidate HIV vaccines that did provide protection against SIV at the pre-clinical stage (which was attributed to ADNKA), were unsuccessful in human clinical trials (107). Contributing to this limitation is the deficiency of species-specific or species-cross-reactive reagents. With that said, murine reagents are widely available and the availability of NHP reagents is improving [NHP-reactive antibody clones are reported on databases such as NIH NHP Reagent Resource (www.nhpreagents.org/_)]. However, the sparser reagent pool and incomplete characterisation of animal model immune components, together limits our ability to yield results that are replicable in human studies.

Furthermore, the scope for genetic manipulation at the NHP level is minimal, particularly in comparison to that of mice, where immunodeficient mice can help us understand the weight of the role of particular immune parameters in mediating protection. Therefore, for NHP-level immune manipulation we rely on immunodepleting with mAbs against specific cellular subsets, or FcyR inhibitors, prior to challenge and rechallenge to define the relevance of cell subsets and Fc effector functions in mediating protection, respectively. However, this approach is not 100% effective, as was observed in CD4+ depletion experiments referenced in (28), which may in some cases be attributable to the limited or less-optimal NHP-reactive reagents. Additionally, for genetic manipulation studies, we must be at a stage to confidently predict candidate CoPs in order to minimise animal sample sizes and unnecessary/wasteful use of research animals.

### 6.2 Advances in human research to assist in the search for CoPs

A combination of human and animal data often yields the greatest insights into a pathogen’s CoP. Furthermore, the best model for human infection is no doubt the human itself. Hence, advances in biotechnology, immunology and human challenge trials must be applied to further improve CoP research.

To delve into immune responses post-vaccination in detail, LN GC reactions are analysed using digestion or microscopic dissection of isolated animal tissue. However, until recently blood biomarkers such as CXCL13 and circulating Tfh cells were relied upon to detect GC reactions in humans (98). The shortcomings of such techniques include the uncertainty of the antigen-specificity of the reactions, and the limited and short traceability of these markers (98). Fine needle aspiration (FNA) has since allowed for the analysis of GC reactions in the ipsilateral axillary draining LNs (IADLN) of 15 humans vaccinated with BNT162b2 or mRNA-1273 (98). Using fluorescently labelled SARS-CoV-2 probes, an amplification of SARS-CoV-2-specific GC B cell, Tfh cell, class-switched MBC and plasma cell frequencies could be observed post-boost in the IADLNs (98). The significance of the development of the FNA technique is exemplified by the fact that circulating Tfh cell populations, that are amplified post-vaccination, did not correlate with IADLN Tfh cells, SARS-CoV-2-specific GC B cells or NAb responses (98). In other words, whilst these peripheral cells are likely indicative of ongoing LN GC reactions, they are not accurate biomarkers of SARS-CoV-2-specific GC B cell, Tfh cells and MBCs in the IADLN and so fail to accurately illustrate GC reactions, thus highlighting a notable place for FNA in human GC research (98). Therefore, the void that FNA will fill in the study of human GC reactions will no doubt contribute to a greater understanding of this node of the immune system and its role in mediating a protective immune response.

Irrespective of the challenges associated with carrying out a human challenge study as discussed previously, the immune response to a species-specific pathogen is best studied within the species of interest. Hence human COVID-19 challenge trials will be invaluable to the field of immunology research. To date, only provisional findings on viral kinetics have been reported by University College London and Imperial College London. Of the 36 young, naïve and unvaccinated participants, 53% became infected upon challenge with 10 TCID_50_ of SARS-CoV-2, 89% of which experienced mild-to-moderate symptoms and the remaining 11% were asymptomatic cases. Only reports on the induction of Spike IgG and NAbs post-challenge have emerged thus far, with future studies aiming to pinpoint the immune parameters providing protection in the 47% that did not become infected following challenge (71).

Additionally, the development of organoid, ‘LN-on-a-chip’ technologies will reduce the demand on research animals, thus providing ethical and logistical solutions to the challenges associated with animal research. An organoid developed in the Singh lab, is a gelatin and silicate nanoparticle-based network, that with the addition of appropriate stimuli including integrins, IL-4 and CD40L, has the capacity to direct the differentiation of GC-like B cells at controlled rates within one week (108, 109). ‘From one mouse spleen, 500 organoids can be generated, or one human tonsil can mediate the synthesis of 1,000 organoids’ (110). This exciting field of research will likely attract extensive interest in coming years and perhaps define the future of immunology.

## 7 Conclusion

Defining pathogen-specific CoPs is a valuable, yet challenging, endeavour for vaccinologists and immunologists. SARS-CoV-2 is now an endemic CoV and will likely persist as another seasonal human coronavirus infection. Therefore, aged or immunocompromised individuals are likely to receive a seasonal vaccination, as is currently advised for influenza virus. However, the difference between SARS-CoV-2 and influenza virus, is the lack of confidence in the SARS-CoV-2 CoP. The absence of a SARS-CoV-2 CoP minimises the capacity for immunobridging, which would support the approval of yearly variant vaccines, thereby slowing the vaccine approval process and putting pressure on vaccine supply networks.

While proposed SARS-CoV-2 CoPs have successfully facilitated immunobridging for the accelerated approval of SARS-CoV-2 vaccines in subgroups of the population who were not included in original human trials (children and pregnant women), and informed ‘boosting’ regimens (111), regulatory agencies remain reluctant to approve vaccines in the absence of an accepted SARS-CoV-2 CoP. Although this is not unheard of when immunogenicity data is directly compared with an approved vaccine, for example, VLA2001 when compared to ChAdOx nCoV-19 (112). Additionally, regulatory agencies recommended approval of the bivalent mRNA-1273.214 (WT/BA.1) based on comparison with approved mRNA-1273 (113), and BNT162b2 Bivalent (WT/BA.4/BA.5) when compared with approved BNT162b2 (114). Acceptance of a SARS-CoV-2 CoP, for which standardised assays have been or can be developed (as is seen with the HAI assay for influenza), will improve the scope for immunobridging, thus accelerating SARS-CoV-2 vaccine approval to meet global demands.

**Figure 6:**
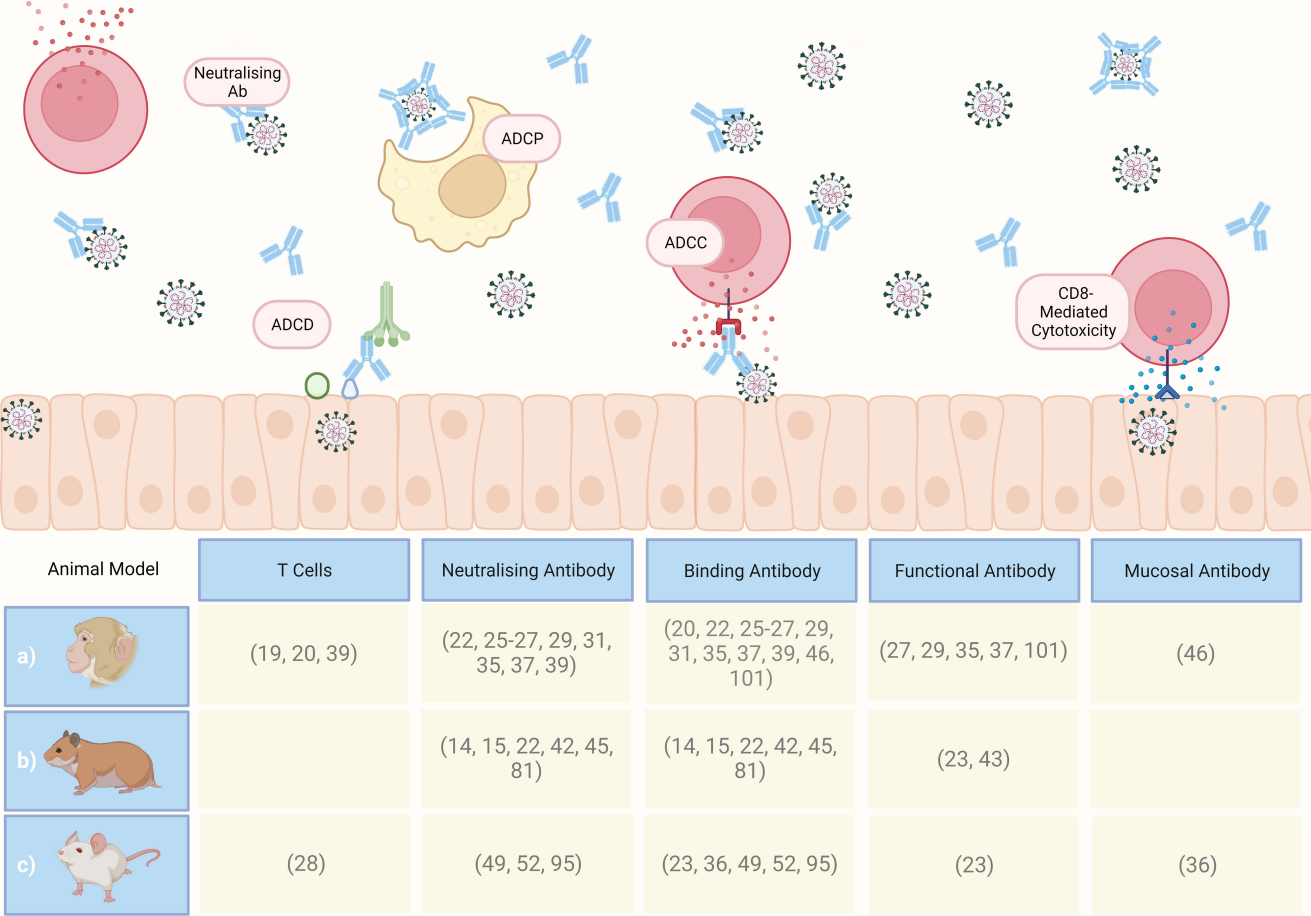
Graphical summary of CoPs proposed in the referenced literature following computational analysis of preclinical study datasets, involving a) macaques, b) Syrian golden hamsters, and c) mice. ADCP, antibody-dependent cellular phagocytosis; ADCC, antibody-dependent cellular cytotoxicity; ADCD, antibody-dependent complement; Ab, antibody. Created with BioRender.com.

Animal models remain a crucial tool for the identification and confirmation of such CoPs, for reasons outlined in this review. As summarised in **Figure 6**, the weight of evidence from *in vivo* studies, supported by clinical trials, provides a degree of confidence that a SARS-CoV-2 humoral response CoP will soon be defined and accepted by both the scientific community and regulators. Designing animal studies to further characterise CoPs, *via* the mechanisms discussed in this review, will expedite CoP research and vaccine development for SARS-CoV-2 and future pathogens.

## Author contributions

CB & MC were responsible for the concept of the review. CB drafted the manuscript. All co-authors reviewed and contributed to the manuscript. All authors contributed to the article and approved the submitted version.
